# Comprehensive Review of RF MEMS Switches in Satellite Communications

**DOI:** 10.3390/s24103135

**Published:** 2024-05-15

**Authors:** Bingqian Shao, Chengjian Lu, Yinjie Xiang, Feixiong Li, Mingxin Song

**Affiliations:** 1 School of Applied Science and Technology, Hainan University, Danzhou 571799, China; 181233@hainanu.edu.cn (B.S.); 20213005464@hainanu.edu.cn (C.L.); 20213005505@hainanu.edu.cn (Y.X.); 20213005499@hainanu.edu.cn (F.L.); 2 School of Electronic Science and Technology, Hainan University, Haikou 570228, China

**Keywords:** RF MEMS switches, satellite communication, reconfigurable antennas, RF performance

## Abstract

The miniaturization and low power consumption characteristics of RF MEMS (Radio Frequency Microelectromechanical System) switches provide new possibilities for the development of microsatellites and nanosatellites, which will play an increasingly important role in future space missions. This paper provides a comprehensive review of RF MEMS switches in satellite communication, detailing their working mechanisms, performance optimization strategies, and applications in reconfigurable antennas. It explores various driving mechanisms (electrostatic, piezoelectric, electromagnetic, thermoelectric) and contact mechanisms (capacitive, ohmic), highlighting their advantages, challenges, and advancements. The paper emphasizes strategies to enhance switch reliability and RF performance, including minimizing the impact of shocks, reducing driving voltage, improving contacts, and appropriate packaging. Finally, it discusses the enormous potential of RF MEMS switches in future satellite communications, addressing their technical advantages, challenges, and the necessity for further research to optimize design and manufacturing for broader applications and increased efficiency in space missions. The research findings of this review can serve as a reference for further design and improvement of RF MEMS switches, which are expected to play a more important role in future aerospace communication systems.

## 1. Introduction

The increasing demand for advanced reconfigurable communication technologies and their products [1] has been accentuated by significant advancements in modern satellite and radar communication fields [2,3]. There is an urgent need for integrated RF communication systems that are low-cost, low-power, reconfigurable in frequency, and capable of multi-band operation [4,5]. MEMS (Microelectromechanical Systems) technology has made revolutionary strides in various fields such as photonics [6], sensors [7], and communications [8], fundamentally altering electronic technology. Particularly in RF applications, the advent of MEMS technology has sparked widespread interest in developing RF devices such as switches [9], phase shifters [10], antennas [11], and filters [12]. Among these, RF MEMS switches, with their miniaturization, low cost, low power consumption, excellent RF performance, and reliability, have found widespread use in radar and satellite communications.

Recent advances in aerospace technology have focused on launching micro- and nanosatellites, each weighing only a few tens of kilograms, into space. In satellite systems, where the launch cost of each kilogram of payload can be hundreds of thousands of dollars, the communications subsystem accounts for over 15% of the satellite’s load. Satellite payloads typically feature hundreds of switches integrated in a matrix form to provide system redundancy. Low-power redundancy switch matrices are usually implemented using coaxial switches [13], which are bulky and undesirable for burgeoning micro- and nanosatellites, necessitating new alternatives to reduce size and weight. RF MEMS technology can be used to construct miniaturized redundancy switch matrices, replacing these bulky coaxial switches.

Semiconductor devices and components, such as PIN diodes and FETs suffer losses at high frequencies despite their numerous advantages [14]. In recent years, atomic-level memristive switches have shown great potential in RF applications due to their high bandwidth and rapid response [15]. However, the development of memristive switches is still in its infancy, and wafer-scale production faces many bottlenecks [16]. Therefore, designing high-linearity, high-efficiency, and miniaturized devices and components has become one of the most challenging tasks in the field of wireless communication systems.

RF MEMS switches offer superior performance over traditional RF switches in microwave applications [17], making them effective for deployment in wireless antenna systems. In satellite communication systems with multiple antennas, RF MEMS switches can be used to select specific antennas for communication, adapting to different transmission requirements and environmental conditions. Their low driving voltage and low power consumption significantly improve the battery life of communication devices, which is crucial for long-term space missions and helps extend the lifespan of satellites. Additionally, the application of RF MEMS switches in satellite uplink and downlink networks, especially in the switching process between frequency allocators and transceivers/receivers [18], demonstrates their broad applicability.

In this review, we investigate the operating mechanisms of RF MEMS switches in the second section, including driving mechanisms (electrostatic, piezoelectric, electromagnetic, and electrothermal) and contact mechanisms (capacitive and ohmic). The advantages and challenges associated with each type are also discussed. The third section focuses on strategies to enhance the reliability and RF performance of RF MEMS switches, exploring various optimization strategies such as reducing stiction effects, lowering driving voltage, improving contact materials and structures, and appropriate packaging. Moreover, the importance of enhancing the equivalent inductance, capacitance ratio, and optimizing serial-parallel configurations for better RF performance is emphasized. In the fourth section, the article explores the application of RF MEMS switches in reconfigurable antennas, highlighting their enormous potential in future satellite communication systems. The existing reviews of RF MEMS switches tend to concentrate on general aspects such as reliability [19], driving voltage [20], and performance parameters [21]. Through a comprehensive analysis of existing research, this paper highlights the significance of RF MEMS switches in enhancing the performance and reliability of satellite communication systems, reducing costs and power consumption, and discusses their potential and challenges in practical applications. Overall, this paper aims to provide an in-depth analysis of the application of RF MEMS switches in the field of satellite communications, revealing their technological advantages and challenges, as well as their key role in future satellite communication systems. To our knowledge, this is the first comprehensive review to holistically examine the improvement of RF MEMS switches through various strategies for better suitability in space applications, offering a reference for the development or improvement of the next generation of RF MEMS switches, particularly in the field of satellite communications.

## 2. Advanced Technologies and Mechanisms in RF MEMS Switches

RF MEMS switches are devices that operate in the RF to the millimeter-wave frequency range, consisting of two distinct parts: the mechanical and the electrical. The mechanical part is driven by one of four actuation mechanisms: electrostatic, piezoelectric, electromagnetic, or electrothermal, facilitating the lateral or vertical movement of the switch. The electrical part is categorized by the contact type, as either capacitive (metal-insulator-metal contact) or ohmic (metal-to-metal contact), and by the circuit configuration, as either series or parallel. The following sections further elaborate on these types of switches and their respective research.

### 2.1. Actuation Mechanism

**Figure 1:**
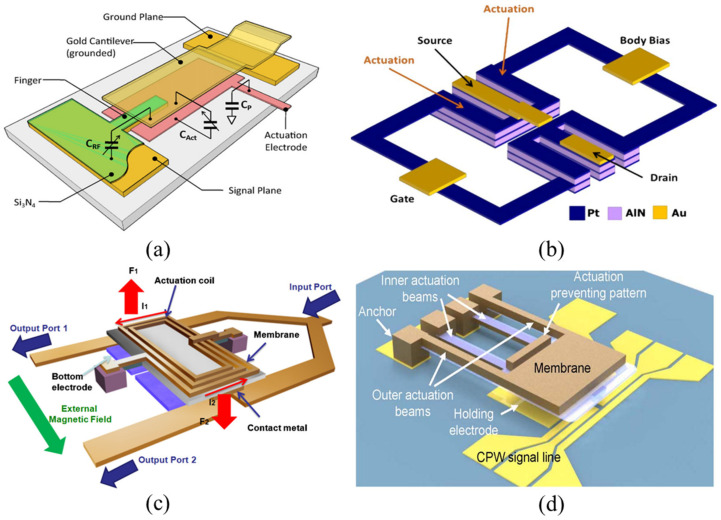
Structures of four conventional RF MEMS switches: (**a**) Electrostatically actuated RF MEMS switch [25]. Reprinted/adapted with permission from [25]. Copyright 2011, with permission from Elsevier. (**b**) Piezoelectrically actuated RF MEMS switch [26]. Reprinted/adapted with permission from [26], 2012, IEEE. (**c**) Electromagnetically actuated RF MEMS switch [27]. Reprinted/adapted with permission from [27]. (**d**) Electrothermally actuated RF MEMS switch [28]. Reprinted/adapted with permission from [28], 2020, IEEE.

RF MEMS switches can be divided into electrostatic, piezoelectric, electromagnetic, and electrothermal based on traditional actuation methods. Figure 1 illustrates the typical structures of these four types of switches.

In space applications, RF MEMS switches or structures, compared to PIN and optical switches, may demonstrate more ideal performance due to their non-involvement with radiation-sensitive electronic components [22]. In terrestrial wireless communication systems, the choice and number of switches depend on the required operational speed and specific requirements of the entire system. Silicon, a common substrate material, shows no significant degradation in mechanical properties even under radiation doses exceeding 100 Mrad. The radiation sensitivity of devices is largely influenced by their actuation mechanism, structural design, and material choice [23,24], primarily related to the disturbance caused by charge trapping in dielectrics under radiation. Notably, electrostatically driven MEMS devices are extremely sensitive to charge accumulation in the dielectric layer. In contrast, MEMS devices using other actuation mechanisms, such as thermal and electromagnetic, exhibit higher radiation resistance.

#### 2.1.1. Electrostatically Actuated RF MEMS Switches

Figure 1a shows the structure of a typical electrostatically driven RF MEMS switch. Among various actuation principles, electrostatic actuation is the most common in RF MEMS switches due to its simplicity, low power consumption, high-frequency circuit compatibility, and good compatibility with CMOS (Complementary Metal–Oxide–Semiconductor) fabrication processes. These switches play a key role in constructing SPDT (Single-Pole Double-Throw) and more complex SPMT (Single-Pole Multiple-Throw) networks. Core performance parameters include the actuation voltage, switch capacitance ratio, on-state insertion loss, and off-state isolation, which are critical metrics for assessing the performance of electrostatic RF MEMS switches.

Despite many advantages, electrostatically driven RF MEMS switches face two main challenges: reducing the actuation voltage and improving mechanical structure stability. High actuation voltages shorten the lifespan of switches and cause failure due to charge trapping in the dielectric layer. A high actuation voltage is impractical for satellite communication applications, as it necessitates the addition of bulky step-up converter circuits. To achieve efficient voltage conversion, step-up converters need to have sufficient inductors and capacitors to store and release energy [29], which necessitates a certain volume for the converter. Smith et al. [30] confirmed through research that, for capacitive RF MEM switches, a reduction of 5–7 V in actuation voltage can extend the switch’s lifespan by 10 years. Shekhar et al. [31] reported a low actuation voltage RF MEMS switch, requiring a very low actuation voltage (4.8 V). After undergoing 10 million switching cycles, its RF performance showed little change, with no failures, proving that a low voltage can effectively prolong the switch’s lifespan.

#### 2.1.2. Piezoelectrically Actuated RF MEMS Switches

Figure 1b shows the structure of a typical piezoelectrically driven RF MEMS switch. In these switches, piezoelectric materials are applied to the top of the beam. Under voltage, these materials produce elongation and strain along the entire piezoelectric layer, thereby driving the beam membrane. The magnitude of the actuating force depends on the piezoelectric coefficient, and low actuation voltages can be achieved by choosing materials with high piezoelectric coefficients [26].

Common piezoelectric materials in MEMS devices are AlN [32] and PZT [33,34]. PZT, a ferroelectric material, has a spontaneous polarization that can be permanently altered by an electric field, i.e., by changing the orientation of dipoles to produce a hysteresis loop to the electric field (or voltage) [35]. Compared to PZT, AlN has the advantage of compatibility with current CMOS processes and lower leakage current at the same film thickness [32]. Additionally, AlN has a lower dielectric constant, meaning lower energy consumption for a given actuator size [26]. Although PZT has a higher piezoelectric coefficient, high-quality, reproducible thin films have always been a technical challenge. Guerre et al. [33] designed and integrated a PZT-based RF MEMS switch into CMOS technology, demonstrating the BEOL (Back End of Line) CMOS compatibility of this integration technique. The switch’s actuation voltage is 15 V, with an insertion loss of less than 0.5 dB and isolation better than 30 dB in the frequency range of 0.4 to 6 GHz.

#### 2.1.3. Electromagnetically Actuated RF MEMS Switches

Figure 1c shows the structure of a typical electromagnetically driven RF MEMS switch. Electromagnetic RF MEMS switches, as a special type of latching switch, are characterized by a low loss, high linearity, and wide bandwidth [36,37]. Niarchos [38] demonstrated that on the microscale, electromagnetic forces dominate over electrostatic forces. The working principle of electromagnetic MEMS switches is based on the electromagnetic interaction between magnetic materials and active (coil) or passive magnetic field sources (permanent magnets). The electromagnetic force drives the movement of the beam membrane when direct current is applied to the coil.

Using permanent magnets or ferromagnetic materials, electromagnetic MEMS switches can achieve a stable latched state without the need for external energy input; i.e., the beam membrane can maintain a fixed position, either up or down. In this case, the magnetomotive force generated by the permanent magnet forms the core part of the magnetic circuit. The magnetic field produced by the coil is opposite to the magnetization direction of the cantilever beam, achieving ON/OFF switching [39]. With the miniaturization of MEMS devices, the required magnetic force decreases. For instance, Glickman et al. [40] designed and fabricated a laterally driven magnetic MEMS switch, which can generate about 200 μN of magnetic closure force under less than 0.7 V driving voltage and 13 mW power.

Although electromagnetic MEMS have clear advantages over electrostatic and piezoelectric actuators in terms of force, polarity, and driving distance on the microscale, they are relatively slower, larger in size, and typically do not use CMOS-compatible manufacturing processes [40]. The compatibility of MEMS technology with magnetic materials is a key area of current research, with many research institutions around the world actively exploring this issue.

#### 2.1.4. Electrothermally Actuated RF MEMS Switches

Figure 1d shows the structure of a typical electrothermally driven RF MEMS switch. Compared to other actuation methods, the primary advantage of electrothermal actuation lies in its lower actuation voltage threshold to initiate switch operations [28].

The operation of electrothermal RF MEMS switches is based on the thermal expansion coefficient of materials. The heat generated by the current flowing through the resistor on top of the beam causes the beam to deflect. This electrothermal actuation method not only produces a stronger force than electrostatic actuation but also achieves high isolation, requiring only a lower actuation voltage (typically a few volts) [41,42]. Additionally, Shojaei-Asanjan et al. [43] conducted a multiphysics analysis of electrothermally driven RF MEMS switches in power limiter applications. The results indicated that compared to their electrostatic counterparts, electrothermal RF MEMS switches offer higher device sensitivity in such applications. However, a major drawback of electrothermal actuation is the relatively larger device structure, leading to increased power consumption during operation. Since electrothermal RF MEMS switches require the application of an actuation current both during the switching and holding phases, they typically consume more electrical energy.

### 2.2. Contact Mechanisms

RF MEMS switches typically rely on two main contact mechanisms: capacitive (i.e., metal-insulator-metal contact) and ohmic (i.e., metal-to-metal contact). Capacitive switches utilize changes in capacitance between metals and insulators to control signals, making them ideal for high-performance signal processing applications such as precision filtering and frequency selection [44]. Ohmic switches, on the other hand, which operate through direct contact between metals, are particularly suitable for handling high-power and fast-switching applications [45]. When selecting RF MEMS switch types, decisions should be based on the specific requirements of the application, considering factors like frequency range, power handling capability, switching speed, isolation, and insertion loss, among other key parameters. Scattering parameters of RF MEMS switches are typically used to assess their RF performance, and they can be theoretically calculated using Equation (1) [46]. When an RF MEMS switch is on, RF signals are attenuated due to factors like series resistance and impedance mismatch, and S_21_ represents the insertion loss, which measures signal transmission efficiency. When the switch is off, RF signals are grounded through the off-state capacitance, and S_21_ indicates isolation, which is the ability to block signals.

(1) $$S_{21}=\frac{2}{2+\omega CZ_{0}}$$

where $\omega =2\pi f_{0}={\left(k/m\right)}^{1/2}$, and the resonance frequency $f_{0}=1/2\pi \sqrt{LC}$. Here, k, m, and L represent the spring constant, effective mass [47], and equivalent inductance of the beam membrane, respectively; C equals $C_{u}$ or $C_{d}$, representing the up-state or down-state capacitance, respectively; and $Z_{0}$ is the switch’s characteristic impedance.

#### 2.2.1. Capacitive RF MEMS Switches

Capacitive RF MEMS switches operate based on capacitive coupling, where the switching of states is achieved by moving the electrical part into or out of contact with the transmission line. Compared to ohmic switches, they are typically used in higher frequency scenarios [48,49] because they have better isolation and lower insertion loss at higher frequencies [50], such as the millimeter wave band (30–300 GHz). In high-frequency applications, the capacitive coupling characteristics of these switches enable them to effectively transmit high-frequency signals while maintaining high isolation and low insertion loss. Additionally, these switches are easier to miniaturize and lightweight, meeting the strict space and weight requirements of high-frequency circuits.

**Figure 2:**
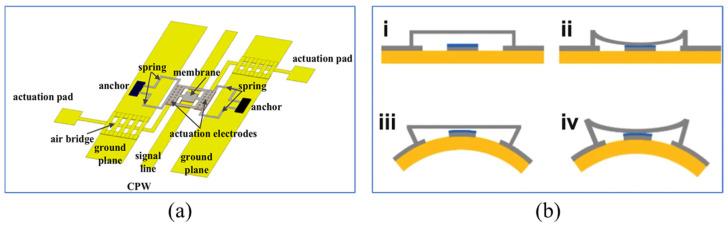
Structures of capacitive RF MEMS switches: (**a**) Li et al. [51]. Reprinted/adapted with permission from [51]. Copyright 2016, with permission from Elsevier. (**b**) Shi et al. [8]. Configuration diagram of flexible RF MEMS switch in different modes: (i) On state and (ii) Off state of MEMS switch with flat substrate; (iii) On state and (iv) Off state of MEMS switch with curved substrate. Reprinted/adapted with permission from [8].

Challenges commonly faced by capacitive MEMS switches include dielectric stiction/breakdown and mechanical impact caused by high actuation voltages, hindering their commercialization. The actuation and capacitive areas of RF MEMS switches can be designed separately [51], as shown in Figure 2a. Such switches can be integrated into RF systems without additional circuits to isolate the DC voltage, cleverly avoiding damage to the system from high actuation voltages.

Flexible RF MEMS switches, with advantages such as conformability, low cost, and impact resistance [52,53,54,55], ensure high-performance, high-integration, and lightweight devices. They are suitable for satellite/aerial radar or IoT communication systems, especially in satellite communication applications where large curvature bending or scalable conformal integration is required. Flexible RF MEMS switches are basic structural units in flexible RF MEMS switch matrices [13,56] and reconfigurable MEMS antennas [57], such as island bridges connecting MEMS units. In the system, each individual switch acts as a node within the island bridge structure [58] and needs to adapt to a certain degree of curvature. Shi et al. [8] designed a flexible RF MEMS switch, as shown in Figure 2b, achieving a certain degree of bending radius through miniaturized electronic components. When the curvature varied from 0 mm^−1^ to 0.10 mm^−1^, the RF characteristics of this switch were insensitive to bending deformation. However, when the switch is in the On state and the curvature exceeds 0.10 mm^−1^, its performance rapidly deteriorates. Currently, it has not been confirmed that flexible RF MEMS can maintain stable RF performance under conditions of significant bending [59,60]. This is mainly due to the difficulty in reconciling the manufacturing processes of MEMS with high performance [61,62,63].

#### 2.2.2. Ohmic RF MEMS Switches

Ohmic RF MEMS switches operate based on resistive tuning, achieving signal path disconnection by physically separating the input and output. Ohmic switches are typically used in low-frequency circuits, as they form an actual electrical connection through metal contact points in the closed state, providing a low-resistance path to ensure signal integrity and minimize loss in low-frequency signals. Since a physical connection is formed in the closed state, ohmic switches maintain good signal integrity, which is crucial for analog signals in low-frequency applications. With special design, series MEMS switches can also achieve good RF performance at frequencies up to 110 GHz [64].

Manufacturing MEMS with superconducting materials, though still in development, has the potential to greatly reduce circuit losses. Attar et al. [65] designed a niobium-based superconducting DC contact RF MEMS switch, using standard superconducting microelectronics (SME) technology and multi-step post-processing. This study validated the feasibility of operating MEMS switches at extremely low temperatures, such as 4K. However, at superconducting temperatures, the increased rigidity of the beam membrane required higher actuation voltages. They then proposed an innovative eight-mask fabrication process, integrating low-temperature superconducting niobium RF circuits with gold-based RF MEMS switches [66]. The research demonstrated significant effects of the adopted gold-based MEMS process on the superconducting properties of niobium by comparing RF performance at room temperature and low temperatures.

**Figure 3:**
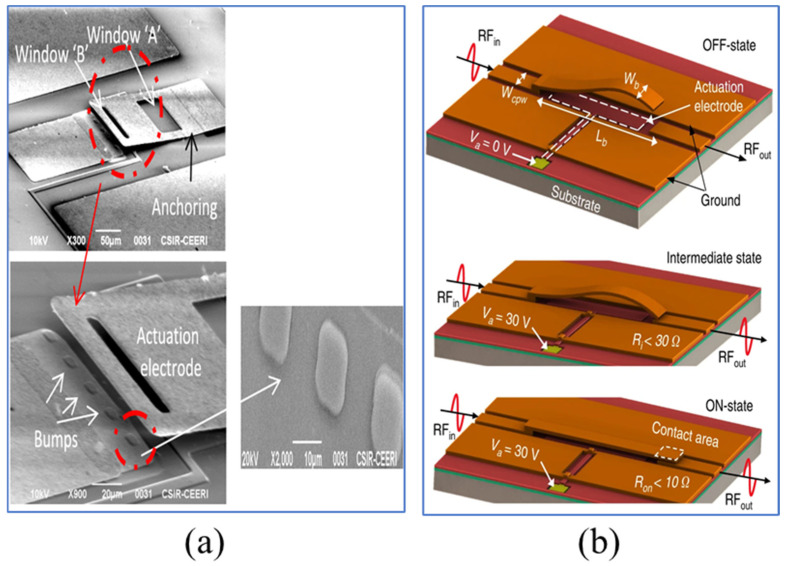
Structures of ohmic RF MEMS switches: (**a**) Bansal et al. [68]. Reprinted/adapted with permission from [68], 2019, IEEE. (**b**) Bajwa et al. [9]. Reprinted/adapted with permission from [9].

Ohmic RF MEMS switches are susceptible to stiction and contact degradation. Minimizing static friction can be achieved by modifying the cantilever [67], but altering the structure requires additional steps, thereby increasing the complexity and cost of the process. Instead of modifying the cantilever beam [68], bumps are introduced in the pull-in electrode manufacturing process. These bumps reduce the contact area by 84%, thus minimizing adhesion significantly. The manufactured switch achieves lower driving voltage with fewer manufacturing steps, as shown in Figure 3a. Traditional MEMS switches require a trade-off between RF performance and driving voltage [20]. Enhancing RF performance, such as by increasing the air gap to reduce insertion loss [69], typically results in an increase in driving voltage. Conversely, lowering the driving voltage may compromise RF performance. Utilizing residual stress in thin films, Bajwa et al. [9] reported an innovative switch design with three-dimensional (3D) wavy microstructures. Figure 3b shows the three states of the switch. This design breaks the limitations of traditional planar cantilever beams, providing additional degrees of design freedom, which can further optimize the switch networks currently used in satellite communications. It utilizes a geometric shape that can be adjusted in three-dimensional space, achieving a lower driving voltage and enhanced RF performance, surpassing conventional flat cantilever switches. However, compared to existing designs [20,70], the downside of this topology is the longer switching time, likely due to its relatively greater length.

## 3. Strategies for Optimizing RF MEMS Switch Performance

A key challenge in the design of space systems is achieving subsystem redundancy to manage component failures. One of the core components of such redundancy architectures is the redundant switch [71], used for routing and switching RF signals between two redundant systems. In the event of a component’s failure, the switch can quickly switch to a backup path or system, ensuring the continuity of communication. Traditionally, these switches have been implemented with bulky and heavy coaxial relays. However, RF MEMS switches, as a promising alternative technology, not only have excellent RF performance but also a smaller size and lighter weight. For space applications, reliability is a crucial consideration. Modern satellites typically require mission lifetimes of several decades [72], thus devices within satellites must maintain long-term stable operation. This is why redundancy designs are often used to reduce the overall system’s failure risk. In aviation, equipment may face temperature ranges from −50 °C to 125 °C, and if installed outside a spacecraft, environmental conditions can be even more extreme.

In satellite communications, RF MEMS switches and devices with excellent RF performance can provide wider bandwidth and higher data transfer rates. This means that enhancing the RF performance of RF MEMS switches can transmit more data, including high-resolution images and videos, significantly improving communication efficiency and quality.

### 3.1. High Reliability

With the continuous increase in performance and reliability requirements of RF systems, developing high-performance switches that can meet complex communication demands has become particularly important. However, the structural characteristics of RF MEMS switches make them prone to failure [73]. In commercial applications, lifespan and long-term reliability have become major limiting factors [74]. Especially in satellite applications, where devices need to maintain stability over many years, the failure time prediction of RF MEMS switches under long-term excitation is critical [75].

Over the past few decades, extensive research has been conducted to address these reliability issues, covering aspects like dielectric charging, mechanical creep, and contact degradation. Different failure mechanisms can affect the performance of switches, which can be categorized into three main types: (1) electrical [76,77,78], including charge trapping and electrostatic discharge; (2) mechanical [79,80,81], mainly involving degradation and creep of contact surfaces; (3) environmental mechanisms [76,82], mainly involving the effects of humidity, environmental particles, and contaminants. Measures such as reducing the impact of shocks, lowering actuation voltages, and improving the materials and structure of contacts can significantly minimize mechanical and electrical degradation. Meanwhile, employing good packaging techniques can effectively control problems related to the environment.

#### 3.1.1. Reducing the Impact of Shocks

In RF MEMS switch applications, significant impacts can accelerate the degradation of contact surfaces and creep, reducing the performance of the dielectric layer [83,84]. This amplifies the likelihood of failure due to adhesion. Increased impact velocity can strengthen contact bouncing and residual vibrations [67,85,86], mainly due to the inertial momentum of the movable electrode and the elastic energy stored in its structure [85,87]. This bouncing can last for an extended period and does not stop until a stable contact is achieved. High-intensity contact impacts can cause electrical discontinuities, material damage, and dielectric charging [86], detrimental to lifespan and reliability. Therefore, taking measures to eliminate or reduce the speed of an electrode bounce to enhance the reliability of these switches is necessary. Various corrective strategies have been developed to mitigate the effects of contact, including open-loop [88] and closed-loop [89] control schemes.

**Figure 4:**
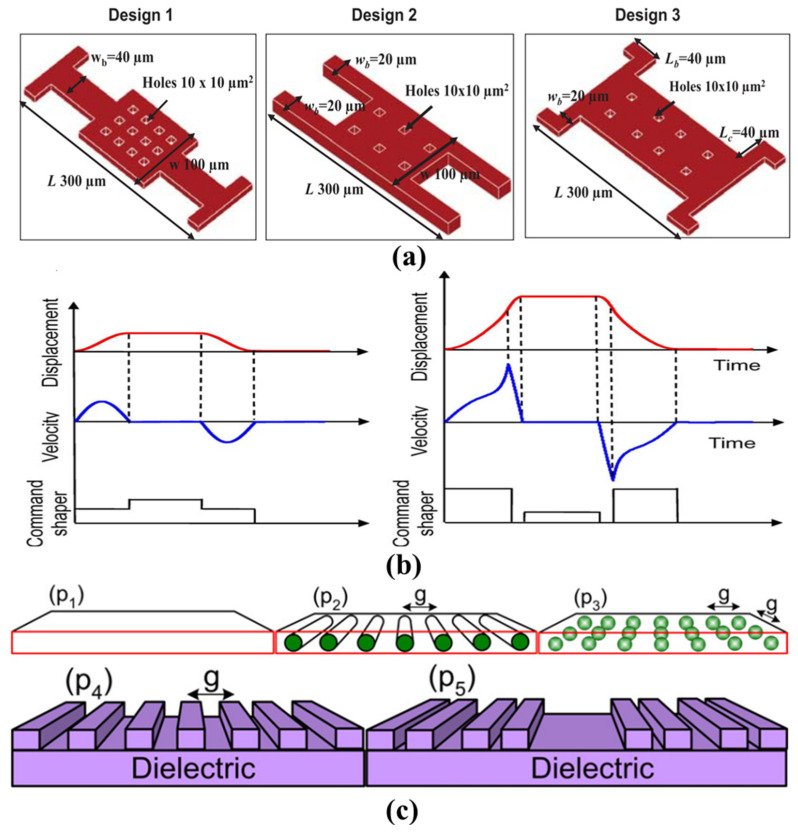
(**a**) Schematics of three designs of capacitive RF MEMS switches. (i) Design 1: T-support, (ii) Design 2: parallel-support, and (iii) Design 3: L-support beams [90]. Reprinted/adapted with permission from [90], 2017, IEEE. (**b**) Illustration of the nonlinear command shapers for electrostatically actuated MEMS systems. (i) Two-step nonlinear shaping method for positioning and (ii) shaper for contact force minimization [93]. Reprinted/adapted with permission from [93], 2011, IEEE. (**c**) Soft-landing by patterning the electrode upper/lower or dielectric. Electrode upper/lower can be a (p1) rectangular plate, (p2) array of cylinders, or (p3) array of spheres, and dielectric can be (p4) an array of linear slots or (p5) a fractal of linear slots [88]. Reprinted from [88], with the permission of AIP Publishing.

The overall dynamic characteristics of the switch can be improved by modifying the electromechanical and electromagnetic modeling of the switch’s geometry, as well as the number and size of its holes. The switches shown in Figure 4a [90] are manufactured on a Pyrex glass substrate using a four-layer mask surface micromachining process. These MEMS switch designs require only a low actuation voltage of 4.8–6.2 V. They achieve bounce-free switching during contact and quickly stabilize after release. Customized pulse drive waveform schemes, widely applied to minimize velocity issues related to contact impact, are used [91,92]. A solution based on command shaping [93], developed through the application of energy conservation, force balance, and elliptical integrals, is shown in Figure 4b. Experimental validation indicates that this approach achieves rapid positioning by suppressing motion-induced vibrations, thus reducing the contact effects on MEMS devices.

However, even minor deviations in parameters may result in significant changes in the force, due to the highly nonlinear characteristics of electrostatic forces, thereby potentially leading to serious collisions. Therefore, the design of excitation pulse waveforms is particularly sensitive to parameter deviations, which might not be the best choice for mass-produced devices. To address this issue, Xiang et al. [89] proposed a novel method of adaptive stiffness. This approach enhances inherent stiffness adaptively in the area near the closed position, improving the robustness of mass-produced switches against parameter deviations. When the actuation voltage is slightly increased, constant stiffness switches convert more electrostatic energy into collision energy for the movable plate compared to adaptive stiffness switches. This new method effectively offsets the significant changes in electrostatic force caused by parameter deviations.

Jain et al. [88] proposed a dynamic soft landing scheme that effectively reduces impact velocity without affecting other performance characteristics such as actuation voltage and actuation time. Figure 4c illustrates the electrode or dielectric patterning design, where the field lines associated with the patterned array begin to rapidly separate as the upper electrode approaches the dielectric during the actuation process. This separation causes the array elements to act as isolated capacitors, rapidly reducing the effective area. Such a decrease in capacitance leads to a reduction in electrostatic potential energy, thereby lowering impact velocity. This technique also helps in reducing dielectric charging, as the contact area decreases, thereby extending the switch’s lifespan.

#### 3.1.2. Reducing Actuation Voltage

Currently, many RF MEMS switches require high actuation voltages, often reaching several tens of volts. This high voltage requirement makes them less practical for applications like mobile wireless communication and reconfigurable circuits. Furthermore, high actuation voltages can lead to unstable dielectric behavior, limiting the switch’s lifespan and reliability [90]. High actuation voltages also cause significant impact forces on the moving beams during contact, increasing the risk of mechanical failure. Additionally, high-impact forces can lead to bouncing and residual vibrations [94,95].

Many researchers are working to reduce the actuation voltage of RF MEMS switches. Studies show that choosing optimal material properties and optimizing switch structural parameters are key to achieving low actuation voltage. The dynamic behavior of MEMS switches is largely influenced by squeeze-film damping, and introducing holes in the structural layer can mitigate squeeze-film effects [96]. Modifying and optimizing beam membrane structure to achieve a low spring constant can significantly reduce actuation voltage but may also reduce beam strength and affect reliability. There is also a trade-off between low spring constant and switch time. For example, reducing air gaps or increasing the area of the electrostatic field drive [97] increases the upstate capacitance, leading to RF signal loss.

Electromagnetic, piezoelectric, and thermally driven MEMS switches have shown potential for low actuation voltages [27,98,99,100]. Although these switches have successfully achieved low actuation voltage, they have disadvantages compared to electrostatically driven switches in terms of higher switch-driving power requirements and structural complexity. To address these issues, researchers have introduced hybrid actuation mechanisms, such as thermal/electrostatic, piezoelectric/electrostatic, and electromagnetic/electrostatic actuation. Table 1 shows some of the performances achieved by switches using these hybrid actuation mechanisms. In these schemes, switches are operated using electromagnetic, piezoelectric, or thermal actuation and maintained in their closed state electrostatically, achieving low-voltage operation and reduced power consumption. However, these schemes also introduce complexities in structure and slower switch speeds.

**Figure 5:**
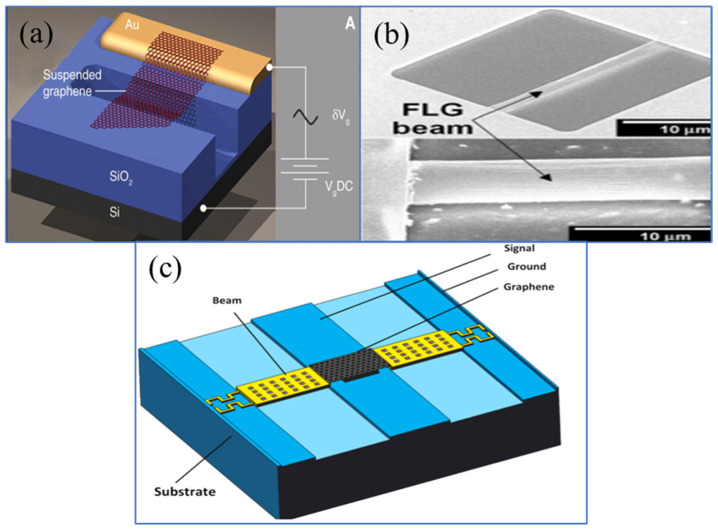
Structures of graphene MEMS devices: (**a**) Bunch et al. [112]. From [112]. Reprinted with permission from AAAS. (**b**) Kim et al. [113]. Reprinted from [113], with the permission of AIP Publishing. (**c**) Zhang et al. [70]. Reprinted from [70]. Copyright 2023, with permission from Elsevier.

Graphene, composed of carbon atoms tightly packed in a two-dimensional honeycomb lattice, exhibits excellent mechanical, thermal, and electrical properties, meeting the requirements for high-reliability contact materials, including high thermal conductivity, electromigration resistance, and fracture resistance. In recent years, graphene has attracted attention for its ultra-thin thickness, high Young’s modulus of 1 TPa, and excellent current carrying capacity of 108 A/cm^2^ [107,108,109]. In the application of NEMS/MEMS switches, graphene has shown its unique electrical and mechanical properties, attracting interest from many researchers. Studies have found that MEMS switches made of graphene can achieve a lower actuation voltage [110] and good RF performance [111]. Bunch et al. [112] first used graphene in MEMS devices. Mechanical exfoliation was used to extract single-layer and multi-layer graphene from graphite and place it on the groove of a SiO_2_/Si substrate to fabricate MEMS resonators, as shown in Figure 5a.

Subsequently, Kim et al. [113] fabricated a graphene beam-type MEMS switch, as shown in Figure 5b. The good mechanical properties of graphene allowed the mechanical switch to operate at an extremely low actuation voltage (1.85 V). Moreover, research shows that increasing the electrical conductivity of graphene can further improve isolation [114], enhancing graphene’s potential application in RF MEMS devices. Zhang et al. [53] designed an RF MEMS switch with a graphene–gold composite upper electrode structure, as shown in Figure 5c. Compared to traditional switches, the actuation voltage was only 3 V, and the isolation of this structure improved by 10 dB. The pioneers’ work indicates that using graphene can effectively solve the high actuation voltage problem of electrostatic MEMS switches, demonstrating graphene’s important role in the future development of MEMS technology. However, current graphene production methods have limitations, especially in the direct acquisition of self-assembled NEMS device structures. For example, the mechanical exfoliation method of separating graphene from bulk graphite is not suitable for the mass production of graphene-based devices. In summary, graphene has clear potential advantages in the application of RF MEMS switch beams, but overcoming challenges in production and integration is necessary for commercialization.

#### 3.1.3. Improvement of Contact Materials and Structures

Contact stiction and degradation are among the most critical failure modes in RF MEMS switches [68]. These reliability issues are closely related to the contact area between metals, especially in ohmic RF MEMS switches. Stiction usually originates from cold welding or hot welding [115]. Cold welding is primarily caused by atomic adhesive forces between contact materials, while hot welding is particularly crucial for switches, as it depends on the current passing through the contact point. This current elevates the temperature, leading to localized welding at the points of micro-roughness.

Ideal contact materials should possess low electrical resistivity, low roughness, high hardness, and high resistance to chemical corrosion and contamination. Gold is a common material for making ohmic switch contacts due to its low resistivity and high chemical corrosion resistance. However, being a soft metal, pure gold contacts often fail to achieve the desired reliability. Therefore, strategies to improve contact life often involve alloying gold with other metals (such as ruthenium, rhodium, or nickel) to increase hardness [116]. However, these strategies have significant limitations, as hardness enhancement is limited at low-alloy metal concentrations, while high concentrations increase resistivity and may introduce brittleness and surface contamination issues. Most metals, when exposed to atmospheric environments, are prone to oxidation or reactions with organics [117], forming non-conductive layers on the surface, which must be removed to establish good electrical contact.

Surface contamination is associated with the presence of reactive metals on contact surfaces that easily oxidize or react with organic materials, forming friction polymers, which lead to a rapid increase in contact resistance [118]. Gold has a significantly lower affinity for forming friction polymers and does not react with common contaminants. From this perspective, the best approach is to increase the hardness of the gold contact points without introducing other metals on the outer surface. Mulloni et al. [115,116] demonstrated that stacking platinum layers between gold not only increased the hardness of the contact points but also improved the contact surface performance. This improved gold-platinum multi-layer contact material significantly outperforms pure gold in terms of stability and shows lower serial resistance. Ideally, understanding the failure mechanisms of MEMS switch surfaces and interfaces requires extensive research under various environmental, temperature, and material conditions to reveal the interactions of various mechanisms and their impact on the overall system response.

**Figure 6:**
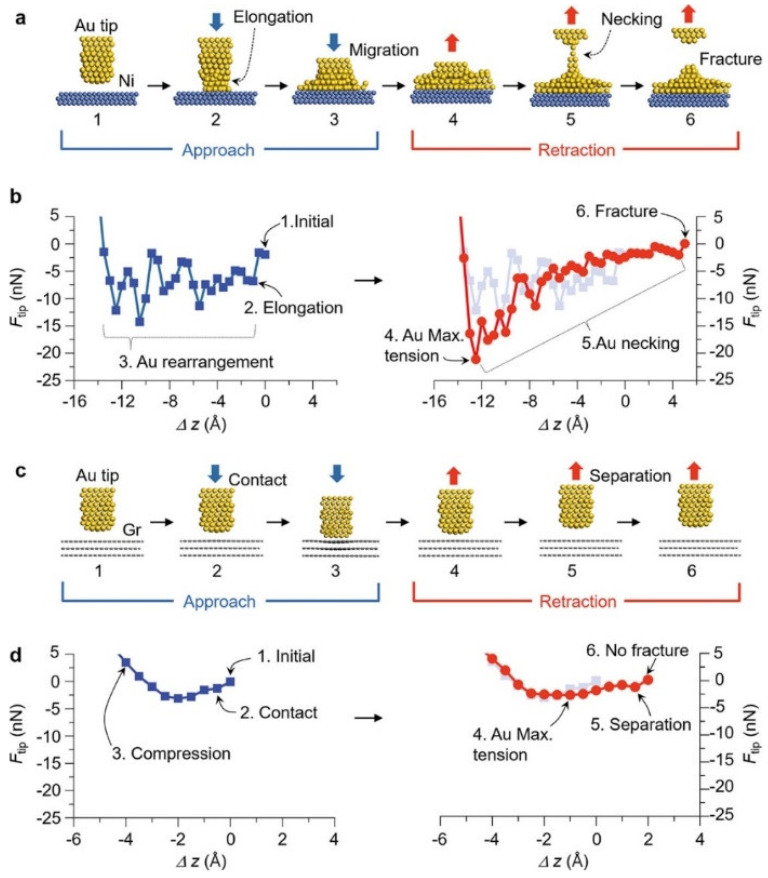
Atomic-scale contact–separation simulation using DFT calculations. [122] (**a**) DFT-optimized atomic structures of the gold (Au) tip and nickel (Ni) surface; (**b**) calculated atomic force of the Au tip, $F_{tip}$ Ftip, during approach (left) and retraction (right). $F_{tip}$ fluctuates considerably and gradually recovers during approach and retraction, respectively, because of the rearrangement of Au atoms, resulting in significant hysteresis; (**c**) DFT-optimized atomic structures of the Au tip and graphene (Gr)-coated surface; (**d**) calculated $F_{tip}$ Ftip during approach (left) and retraction (right). Reprinted/adapted with permission from [122].

Recent advancements in CVD (Chemical Vapor Deposition) technology [119,120] have made it possible to precisely control the morphology and structure of high-quality graphene, leading to its application in various micro- and nanodevices [121]. Seo et al. [122] evaluated graphene as a reliability-enhancing contact material for M/NEM switch devices. Atomic force microscopy experiments and quantum mechanical calculations showed that using a few layers of graphene as the contact material on nickel surfaces can achieve energy-saving features of mechanical contact separation. Compared to bare nickel surfaces, the graphene coating reduced energy dissipation by 96.6%. The DFT (Density Functional Theory) optimized atomic structures and their F-z (force-displacement) curves during the contact separation process on nickel and graphene substrates indicate that graphene almost achieves elastic contact separation, i.e., almost no wear at the atomic scale, including plastic deformation, fracture, and atomic-level wear, as shown in Figure 6. Additionally, under thermal switch conditions, the graphene coating significantly extended the switch’s lifespan, reaching 1000 times that of uncoated switches.

**Figure 7:**
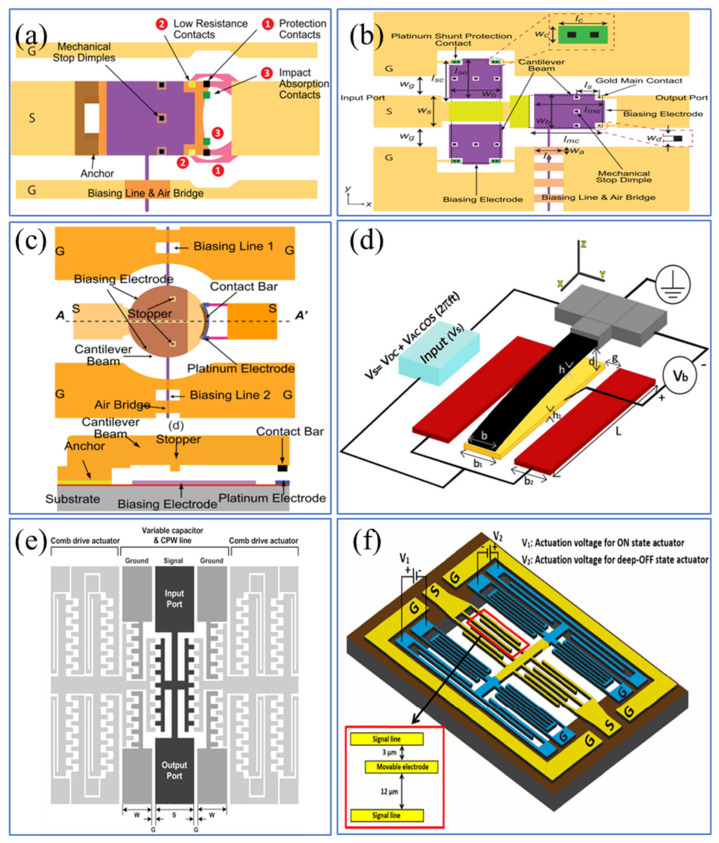
(**a**) Top view of switch with series protection contact [123]. Reprinted/adapted with permission from [123], 2016, IEEE. (**b**) Top view of the shunt-protected switch [124]. Reprinted/adapted with permission from [124], 2017, IEEE. (**c**) The structure of the compact single-cantilever multicontact switch [125]. Reprinted/adapted with permission from [125], 2018, IEEE. (**d**) The structure of levitation-based micro-switch [126]. Reproduced with permission from Springer Nature. (**e**) The structure of a non-contact-type switch [127]. Reprinted/adapted with permission from [127], 2009, IEEE. (**f**) The structure of a three-state contactless switch [128]. The inset shows the initial gap between the signal lines and grounded movable electrodes. Reprinted/adapted with permission from [128], 2015, IEEE.

Novel structural designs can also prolong the lifespan of switches by reducing contact stiction, contact degradation, and charge injection, beyond improving the materials of the contacts [73,92]. The innovative design shown in Figure 7a [123] utilizes a method of paralleling sacrificial contacts with low-resistance contacts, significantly reducing the electric field strength and thereby minimizing contact degradation associated with field-induced material transfer. This design allows the use of a single actuator and bias electrode to implement the correct protective driving sequence, resulting in more than a 100-fold increase in thermal switch lifespan compared to unprotected switches. Liu et al. [124] introduced a parallel protection technique. Protective contacts are placed before the main contact points of the RF MEMS metal contact switch to block the RF signal when the main contact operates, as depicted in Figure 7b. This method creates a local cold switch condition for the main contact, thereby extending the switch’s lifespan under thermal conditions.

Figure 7c [125] presents an innovative cantilever and electrode bias design that integrates multiple contact points within a single cantilever, aimed at enhancing the reliability of the switch. These contact points are used to sequentially conduct RF current until one of them fails. By alternating contact points, the device can refresh and reduce the contact resistance of the switch, thus improving its overall lifespan. Traditional actuation relies solely on reducing voltage to detach the movable electrode (i.e., opening the switch), but any degradation of the contact surface may cause changes in switch characteristics, such as permanent collapse due to strong surface adhesion. The design studied by Mousavi et al. [126], as shown in Figure 7d, employs twoside electrodes to operate a normally closed switch. This design incorporates repulsion auxiliary excitation into the traditional gap-closing capacitor design, overcoming the stiction caused by adhesion forces and providing better switch control. Even with surface degradation, switch operation can be achieved by increasing the side voltage, although this may necessitate higher side voltages.

Further, to avoid common stiction and charge injection issues in contact RF MEMS switches, a design with no physical contact between the signal line and the movable electrode can be implemented by separating the DC bias from the RF signal path. A non-contact RF MEMS switch for radar applications has been reported, as shown in Figure 7e [127]. Subsequently, Pal et al. [128] designed a new type of three-state non-contact RF MEMS switch. Figure 7f displays the structure of the non-contact switch, which is based on a variable capacitance between the signal line and a movable ground electrode controlled by an electrostatic actuator. The proposed switch does not require any ohmic contacts prone to stiction or dielectric layers susceptible to dielectric charging, thus enhancing the device’s reliability.

#### 3.1.4. Using Appropriate Packaging

Operational environmental factors like temperature, humidity, and particulate matter in the environment significantly affect the reliability of switchable devices [19,129,130]. This is particularly challenging in space applications, where devices must withstand extreme temperature variations, humidity fluctuations, or high levels of radiation [24], while still fulfilling their intended mission lifespans. In this context, the packaging of RF MEMS becomes crucial for ensuring their reliability and a high degree of integration. Consequently, researchers have developed multiple technologies to ensure MEMS switches maintain their performance in harsh environments.

MEMS switches require hermetically sealed packaging to prevent any residual moisture or organic contaminants from affecting the internal moving parts. Comprehensive studies are needed on the reliability and electrical performance of devices under different voltage levels and various noise conditions, such as high temperatures and humidity. Understanding the failure mechanisms of RF switches in harsh environments, and their electrical performance under different input frequencies, control voltages, and power levels, remains a key issue to be addressed.

**Figure 8:**
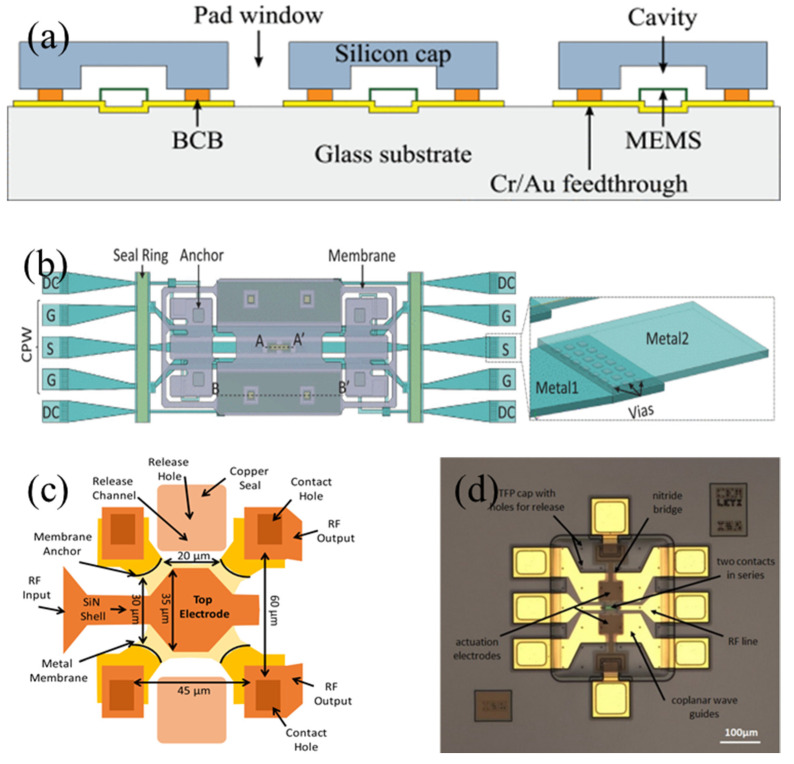
(**a**) Proposed wafer-level packaging approach for RF MEMS devices using BCB bonding [131]. Reprinted/adapted with permission from [131], 2019, IEEE. (**b**) Schematic of the ohmic RF MEMS with a zoom on vias connecting the CPW line to the probing pads outside the seal ring [135]. Reprinted/adapted with permission from [135], 2022, IEEE. (**c**) Layout and dimensions of the zero-level packaged RF MEMS switched capacitors [136]. Reprinted/adapted with permission from [136], 2020, IEEE. (**d**) Top view of a SPST switch including TFP encapsulation [72]. Reprinted/adapted with permission from [72], 2017, IEEE.

WLP (wafer-level packaging) technology is one of the most promising methods for packaging MEMS devices due to its high performance, ease of integration into semiconductor foundry processes, and low cost. Comart et al. [131] proposed a novel packaging method using BCB (benzo-cyclo-butene) as an adhesive layer for encapsulating shunt capacitive RF MEMS switches, as shown in Figure 8a. This packaging method, applied to 50 Ω CPW transmission lines, underwent microwave characterization through circuit modeling. Based on the microwave characterization results, a packaging structure for the shunt capacitive contact of RF MEMS switches was designed, achieving a fault-free lifespan of up to 10.2 billion cycles. Al-Ge eutectic bonding is a widely used bonding technique in microelectronics [132,133], offering significant advantages over other eutectic wafer bonding processes, like Au−Ge and Au−Si, in terms of compatibility with standard CMOS wafer manufacturing processes [134]. Al-Ge eutectic bonding provides not only a conductive path between two substrates but also allows for patterning. Najah et al. [135] successfully integrated Au-Ru/AlCu contacts into the MEMS manufacturing process using Al-Ge eutectic bonding for wafer-level packaging, as depicted in Figure 8b.

WLP allows for the creation of sealed cavities within MEMS-specific process flows, providing robust protection against external environments. However, the resultant component thickness from this packaging method may pose integration challenges with low-cost RF PCB (printed circuit board) subsystems, especially at millimeter-wave frequencies. The addition of a sealing ring around the chip during the packaging process not only increases the overall size of the component but also adds to the losses. In response to this challenge, an alternative method is TFP (thin-film packaging), integrated directly into the MEMS manufacturing process. This method significantly reduces the final component cost and substantially enhances the reliability of such RF MEMS. Figure 8c shows a zero-level packaged MEMS switch capacitor [136]. This minimized packaging is achieved through standard microelectronic process steps within the production flow. Its hermeticity was verified through simple testing, and the device could withstand subsequent PECVD (Plasma-Enhanced Chemical Vapor Deposition) processes for long-term protection. The packaging shown in Figure 8d is also achieved at the wafer level through thin-film packaging technology [72], minimizing the device footprint and being compatible with wire bonding and flip-chip assembly techniques. Moreover, this type of packaged switch exhibits good microwave performance and reliability, particularly in meeting redundancy requirements in satellite communications.

### 3.2. Excellent RF Performance

In communication systems and test instruments, the superior RF performance of RF MEMS switches is reflected in two key indicators: low insertion loss and high isolation. These two factors are critical metrics for evaluating the performance of devices and systems based on RF MEMS switches [137]. High insertion loss can lead to significant RF signal loss, while low isolation may result in signal leakage, both severely affecting the overall performance of the system. Therefore, ensuring RF MEMS switches have a low insertion loss and high isolation is of paramount importance.

#### 3.2.1. Improving Equivalent Inductance

The design and structure of the capacitive MEMS switch beam membrane can be equivalent to a series combination of capacitors, inductors, and resistors [138,139,140]. The equivalent inductance of the switch significantly impacts its RF performance. Therefore, optimizing the equivalent inductance can effectively improve the switch’s RF performance.

**Figure 9:**
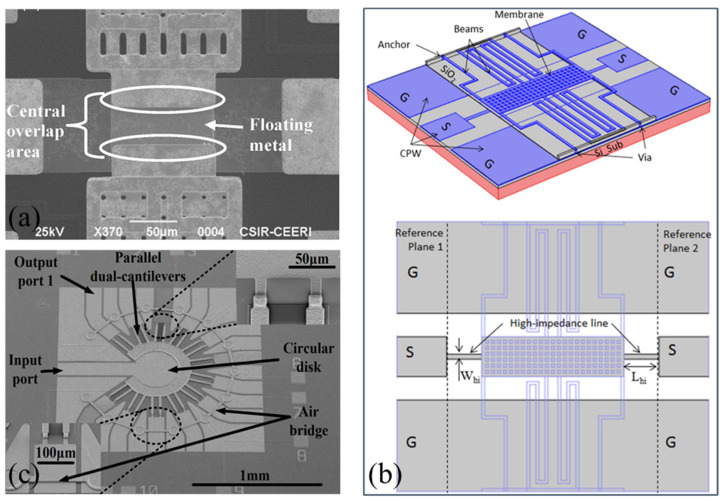
(**a**) The frequency reconfigurable capacitive shunt RF MEMES switch [142]. Reprinted/adapted with permission from [142]. Copyright 2019, with permission from Elsevier. (**b**) The T-match RF MEMS capacitive switch [143]. Reproduced with permission from Springer Nature. (**c**) The SP12T RF MEMS Switch [145]. Reprinted/adapted with permission from [145], 2018, IEEE.

For instance, improving the equivalent inductance of the beam membrane primarily involves adjusting its physical dimensions, shape, materials, and layout. These changes can directly impact the electromagnetic parameters of the switch, including inductance [141], thereby optimizing its RF performance. Angira et al. [142] proposed a capacitive MEMS switch based on two different shapes of non-uniform cantilevers, as shown in Figure 9a. The different structural designs of the cantilever beams can achieve different inductance values when the device is closed, thus adjusting the electrical resonance frequency. Zhang et al. [141] achieved less than a 2 dB low insertion loss in the 240 to 280 GHz range by reducing the equivalent parallel inductance of the switch.

Figure 9b shows an RF MEMS switch with short high-impedance transmission line (T-line) sections added to both sides of the beam membrane [143]. These parts act as series inductors to compensate for the capacitance when the switch is in the upstate. This design provides good matching at the desired frequency, thus improving the isolation by up to 24.05%, at the cost of increased insertion loss. The independence of device impedance from input RF signal power, which indicates linearity, is typically characterized by the third-order intercept point (IIP3) in dual-tone RF intermodulation measurements [144]. For instance, to prevent intermodulation between GSM at 1800 MHz and LTE at 2400 MHz, the IIP3 of the antenna switch must be as high as possible. Zhang et al. [145] designed a novel single-pole twelve-throw RF MEMS switch structure, as shown in Figure 9c. This design uses specially designed parallel double cantilever beams, aimed at improving RF matching and achieving smaller size and more compact devices. The switch also uses the least squares method to reduce the uncertainty of the IIP3 measurement. In this design, the insertion loss and isolation of each channel of the switch are better than 0.94 dB and 30 dB, respectively.

#### 3.2.2. Increasing Capacitance Ratio

In capacitive MEMS switches, the ON/OFF states are achieved by changing the capacitance between the MEMS bridge and the transmission line. According to the previous Equation (1), a small up-state capacitance helps to reduce insertion loss, while a large down-state capacitance aids in increasing isolation. Therefore, the capacitance ratio (C_r_) directly affects the RF response of the switch. A higher capacitance ratio is desired for better RF response [142,146], making it one of the key methods to optimize the RF performance of capacitive MEMS switches. Theoretically, the capacitance ratio of the switch can be calculated using Equation (2) [142]. The equation shows that the capacitance ratio can be effectively increased by using materials with a high dielectric constant, reducing the thickness of the dielectric layer, and increasing the air gap between the electrodes.

(2) $$C_{r}=\frac{C_{d}}{C_{u}}\approx \frac{g_{0}\varepsilon_{r}}{t_{d}}$$

where $g_{0}$ is the air gap between two electrodes; $\varepsilon_{r}$ and $t_{d}$ are the permittivity and thickness of the dielectric layer, respectively; $C_{d}$ and $C_{u}$ are the down-state and up-state capacitance, respectively.

Researchers have explored using materials with high dielectric constants to increase the capacitance ratio. High dielectric constant materials can enhance the RF performance of the switch, but the reliability of the dielectric must be carefully analyzed. Si3N4 (silicon nitride), with its moderate dielectric constant, has been the most commonly used dielectric material for capacitive RF MEMS switches [147]. Approaches using high dielectric constant materials like HfO_2_ (hafnium dioxide) [148,149], AlN (aluminum nitride) [147], Ta_2_O_5_ (tantalum pentoxide) [150], and STO (strontium titanate oxide) [151] have been proposed to achieve high capacitance ratios in MEMS switches. Table 2 shows the RF performance and capacitance ratios achieved with these materials.

The reliability of capacitive RF MEMS switches is limited by leakage current and charging phenomena in the dielectric layer. A common technique to increase the capacitance ratio is to enlarge the air gap between the beam membrane and the dielectric layer, but this method increases the drive voltage, and changing the height of the beam also affects the mechanical characteristics of the RF MEMS switch. Additionally, reducing the dielectric thickness and increasing the dielectric constant are limited due to dielectric breakdown and charge-trapping effects.

**Figure 10:**
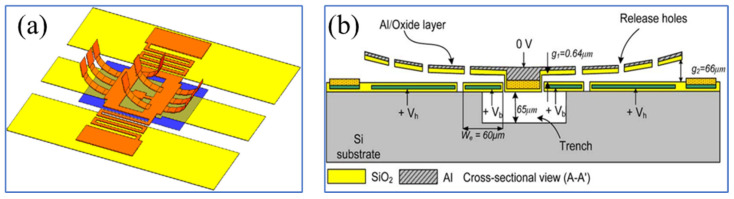
(**a**) The dual-warped-beam switch [152]. Reprinted/adapted with permission from [152], 2010, IEEE. (**b**) The composite metal-dielectric warped membranes [153]. Reprinted/adapted with permission from [153], 2009, IEEE.

Besides the above methods, the capacitance ratio can also be improved by changing the structure of the MEMS switch, such as the double-buckling beam switch shown in Figure 10a [152]. This design increases the effective capacitance area in the downward state by introducing buckling bimetal beams in the switch. As a result, this switch achieves a capacitance ratio of up to 170 without thin dielectrics or high dielectric constant materials and exhibits good RF performance. The switch shown in Figure 10b, consisting of a composite metal-dielectric buckling membrane [153], uses a buckling plate structure to decrease the upstate capacitance and increase the downstate capacitance, achieving a capacitance ratio of 91:1.

#### 3.2.3. Using Serial-Parallel Configurations

Employing serial-parallel configurations, a common method to enhance RF performance in traditional RF switches like FETs, is also applicable to RF MEMS technology, including capacitive and metal contact switches. This structure offers improved RF performance [47,154,155] but at the cost of increased design and manufacturing complexity. For instance, to ensure the optimal performance of an SP4T switch before its application in a phase shifter array, its isolation can be enhanced using a series-type shunt topology at each output port [156].

**Figure 11:**
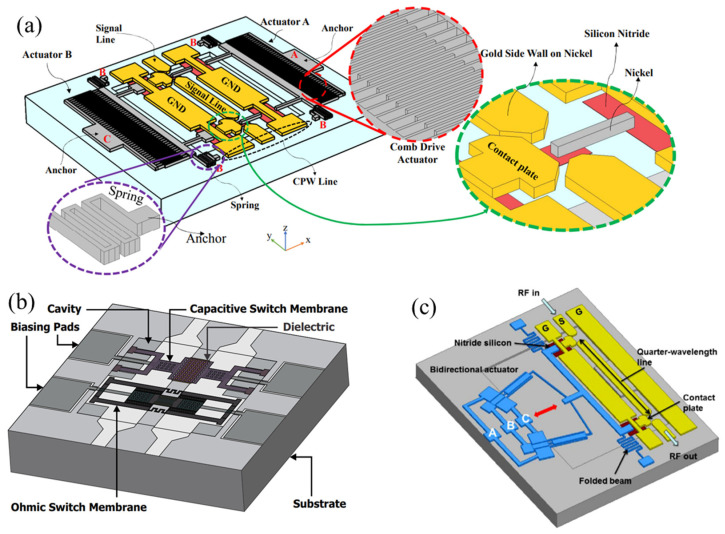
A 3D schematic view of serial-shunt switches: (**a**) Khodapanahandeh et al. [157]. A, B, and C represent different pads, and the state of the switch is controlled by adjusting their voltages. Reprinted/adapted with permission from [157], 2022, IEEE. (**b**) Singh [158]. Reproduced with permission from Springer Nature. (**c**) Zhu et al. [42]. Reprinted/adapted with permission from [42]. Copyright 2014, with permission from Elsevier.

For example, Khodapanahandeh et al. [157] proposed an electrostatically actuated serial-parallel RF microelectromechanical system switch, as shown in Figure 11a. This switch has ON, OFF, and Deep-OFF states and is normally in the OFF state, driven to the ON or DEEP-OFF states using two electrostatic actuators. This switch shows an insertion loss of 0.6 dB and an isolation of 65 dB at 20 GHz, suitable for low-power and high-performance RF tuning. The switch shown in Figure 11b [158] achieves high isolation while maintaining a compact switch size by combining two serial and parallel configurations of thin films on a single substrate. Both capacitive and ohmic beam membranes can be electrostatically driven at the same voltage level. The voltage provided to the parallel beam membrane closes the switch, while the voltage provided to the serial beam membrane opens the switch. However, there are two issues with these serial shunt switches. First, the serial or parallel switch structures need individual biasing, which is difficult to design. Secondly, as they are vertically driven, they require higher driving voltages and thus are unsuitable for many low-voltage applications. The single thermally actuated lateral contact switch shown in Figure 11c is a good alternative [42], as this design simplifies the driving mechanism and reduces the required voltage level.

## 4. Application in Reconfigurable Antennas

**Figure 12:**
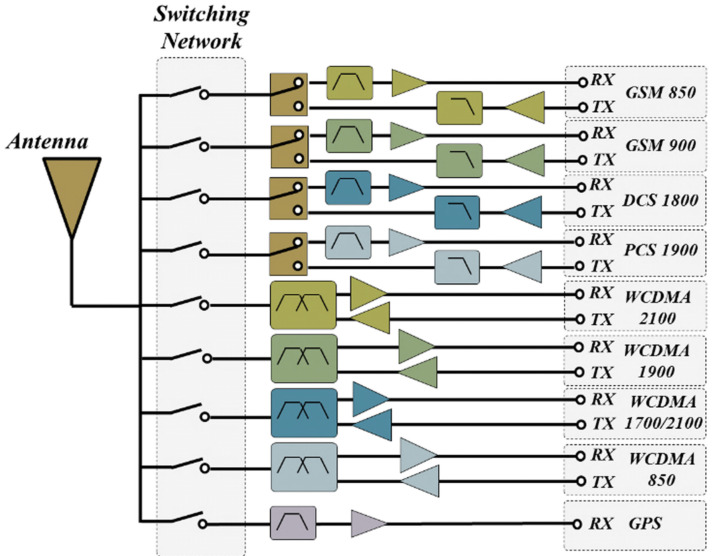
An example of a transceiver that supports different frequency bands [159]. Reprinted/adapted with permission from Ref [159], 2022, John Wiley and Sons.

The miniaturization of satellite communication systems can be achieved by using reconfigurable antennas to reduce the number of antennas in a device. Today, multimode and multi-band RF front-end transceivers are receiving great attention [159]. This is because they can support multiple communication standards, which helps in reducing power consumption, lowering costs, and minimizing signal loss. Figure 12 shows an example of a front-end transceiver that supports GSM/WCDMA/GPS. From the figure, it is evident that the switching network follows immediately after the antenna to enable sharing of the antenna across different frequency bands. The antenna switching network, composed of antenna switches, plays a key role in the overall performance of the multimode modulated front end.

The development of the Internet of Things brings new requirements for antennas in wireless communication systems, especially in areas such as smart cities, smart transportation, and smart healthcare [160,161]. These applications require antennas to be reconfigurable in frequency, polarization, and radiation direction. RAs (reconfigurable antennas) change their effective aperture of the radiation field by rearranging antenna currents or reconfiguring radiating edges, thereby altering the antenna’s characteristics [162,163,164,165,166]. The characteristics of such antennas can be altered by changing the operating frequency, radiation pattern, or polarization behavior. Compared to traditional multi-antenna systems, reconfigurable antennas can significantly reduce hardware complexity and costs while maximally improving antenna performance, all while meeting the ever-changing operational requirements. Therefore, reconfigurable antennas are receiving great attention in fields such as satellite communication [164,165,167,168]. A reconfigurable antenna can change its operating frequency, radiation pattern, and directivity according to different application needs, thus offering greater flexibility and efficiency.

RF MEMS switches are widely used in satellite reconfigurable antennas, enabling reconfigurable multi-band antenna devices based on RF MEMS switches to be reconfigured step by step in microseconds to adapt to a variety of applications in different frequency ranges. Compared to traditional antennas, antennas integrated with RF MEMS switches can reduce parasitic effects and power loss [169]. For example, in phased array antennas, RF MEMS switches can be used to adjust the phase of signals, thus controlling the direction of the beam. This allows satellites to perform more precise beamforming and directional transmission, enhancing communication quality and coverage. Sharma and Gupta [170] described various design parameters to improve the performance of RF MEMS switches, which ultimately enhances the performance of beam-scanning and reconfigurable antennas. It is evident that the performance of antennas largely depends on the design and functionality of RF MEMS switches [171]. The application of these switches not only enhances the flexibility and efficiency of antennas but also helps meet the growing wireless communication needs of IoT.

**Figure 13:**
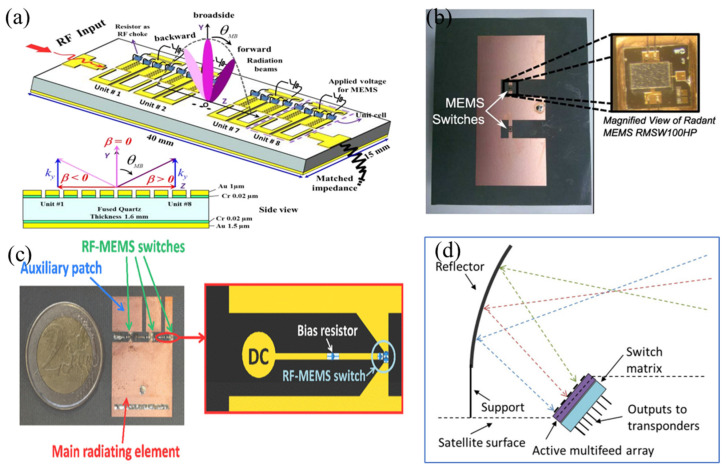
(**a**) The MEMS-modulated scanning antenna [174]. Reprinted/adapted with permission from [174], 2016, IEEE. (**b**) Optimized E-shaped patch antenna with RF MEMS switches [175]. Reprinted/adapted with permission from [175], 2014, IEEE. (**c**) Photograph of the antenna and its MEMS biasing network [178]. Reprinted/adapted with permission from [178], 2016, IEEE. (**d**) The novel multiple-beam antenna for satellite applications [168]. Reproduced courtesy of the Electromagnetics Academy.

The advancement in antenna technology is becoming crucial, especially in ensuring fixed frequency and avoiding RF blockage [172] or interference from multiple RF sources [173]. Figure 13a shows a MEMS-modulated scanning beam metamaterial antenna based on surface micromachining processes [174]. The antenna achieves an electromagnetic homogeneous waveguide with periodic metamaterial modules of unit lengths less than $\lambda_{g}/5$. MEMS switches are integrated into each unit to modulate the phase constant β, thus achieving scanning beams at a fixed frequency of around 8 GHz. Patch antennas are another widely used antenna type in wireless communication, particularly in base stations. However, their application in cognitive radio systems is limited due to their narrow bandwidth. Rajagopalan et al. [175] provided a method using particle swarm optimization to design a reconfigurable antenna with RF MEMS switches for potential applications in cognitive radio systems. By using MEMS switches shown in Figure 13b to dynamically change the slot size, a bandwidth nearly double that of patch antennas can be achieved. The linearity of antennas is also very important for the successful operation of tunable front-ends, as signal correction is not possible after data transmission [176,177]. Although there have been many demonstrations of frequency-tunable antennas in the past, there has been relatively less research in terms of linearity measurements and data transmission capabilities. Chaabane et al. [178] used a switch-assisted band in a compact planar inverted F antenna (PIFA), with the antenna’s center frequency tunable between 1.52 GHz and 2.25 GHz. The RF MEMS switch shown in Figure 13c is used to connect and disconnect the auxiliary band, thereby maintaining power handling capability and antenna linearity.

Additionally, antenna technology for LEO (low Earth orbit) satellites is also progressing [179,180]. Recently launched LEO satellites for Earth observation are equipped with advanced scientific instruments to gather a large amount of data. However, high-speed transmission of all collected data to Earth has become a challenge, mainly due to the relatively short time a single ground station can receive data from a transiting LEO satellite. The new multi-beam antenna shown in Figure 13d [168] can be used for a transparent high-speed data relay system. This system connects multiple LEO satellites to a single ground station through a satellite located in the geostationary orbit (GEO). The antenna switches between different sub-arrays of a larger multi-feed array using a reconfigurable switch matrix based on RF MEMS switches, to track LEO satellites. By combining digital beamforming with beam switching, the radiating characteristics of the antenna can be further improved [181,182].

## 5. Conclusions

This article comprehensively explores the application of RF MEMS switches in satellite communication systems and deeply analyzes how they effectively solve key issues present in current systems, such as weight [183,184], cost [185,186], and power consumption [187,188,189,190]. We find that RF MEMS switches not only provide higher performance and flexibility but also significantly reduce the overall weight of the system, which is crucial for space missions. Additionally, the miniaturization and low power consumption characteristics of these switches open up new possibilities for the development of microsatellites and nanosatellites, which will play an increasingly important role in future space missions.

RF MEMS switches still face some challenges and limitations [118,191,192,193], despite their great potential in satellite communications [194,195]. For instance, the reliability and RF performance of these switches in extreme environments still require further research and improvement. Moreover, to make these switches more common and practical in future satellite communication systems, it is necessary to further optimize their design and address the technical and cost issues in large-scale production. At the same time, advancements in materials science to tackle the current limitations of RF MEMS switches could lead to the development of switches that are more radiation-resistant and have longer lifespans.

In conclusion, RF MEMS switches, as an advanced technological solution, provide unprecedented performance enhancements for satellite communication systems. However, to fully utilize the potential of these switches, we must continue to explore the optimization of their design and how to overcome the limitations of existing technology. Future research should focus on improving the reliability and RF performance of the switches, thereby enhancing the performance and reliability of satellite communication systems, while exploring more economical and efficient manufacturing methods. With these challenges addressed, RF MEMS switches are expected to play an even more important role in future aerospace communication systems.

## Figures and Tables

**Table 1:** Performance of hybrid actuation mechanism RF MEMS switches.

Actuation Mechanism	Actuation Direction	Actuation Voltage	Insertion Loss	Isolation	Ref
Thermal/Electrostatic	Vertical	0.3 V	0.23 dB @ 2.4 GHz	38.80 dB @ 2.4 GHz	[28]
Thermal/Electrostatic	Lateral	7 V	−0.73 dB @ 6 GHz	−46 dB @ 6 GHz	[101]
Thermal/Electrostatic	Lateral	6 V	>−1 dB @ 150 GHz	<−20 dB @ 16 GHz	[41]
Thermal/Electrostatic	Vertical	25 V	1.67 dB @ 5.4 GHz	33 dB @ 5.4 GHz	[102]
Thermal/Electrostatic	Lateral	0.9 V	>−0.1 dB @ 40 GHz	−20 dB @ 100 GHz	[103]
Thermal/Electrostatic	Lateral	0.16 V	<1.2 dB @ 40 GHz	−60 dB @ 35 GHz	[42]
Thermal/Electrostatic	Lateral	0.07 V	−0.27 dB @ 10 GHz	−40 dB @ 10 GHz	[99]
Thermal/Electrostatic	Lateral	0.07 V	>−0.5 dB @ 40 GHz	<−30 dB @ 40 GHz	[104]
Electromagnetic/Electrostatic	Vertical	3.7 V	−0.37 dB @ 20 GHz	−34 dB @ 20 GHz	[105]
Electromagnetic/Electrostatic	Vertical	4.3 V	−0.52 dB @ 20 GHz	−36 dB @ 20 GHz	[27]
Electromagnetic/Electrostatic	Vertical	15 V	0.06 dB @ 14 GHz	50 dB @ 14GHz	[39]
Piezoelectric/Electrostatic	Vertical	5 V	−2 dB @ 20 GHz	−12 dB @ 20 GHz	[106]
Piezoelectric/Electrostatic	Vertical	5 V	<0.5 dB @ 6 GHz	>30 dB @ 6 GHz	[33]

**Table 2:** RF performance comparisons of using different high dielectric constant materials.

Material	$\varepsilon_{r}$	Insertion Loss (dB)	Isolation (dB)	$C_{r}$	Ref
Si_3_N_4_	7.5	−0.90 dB @ 40GHz	−23.7 dB @ 40GHz	22.6	[147]
HfO_2_	25	−0.20 dB @ 20 GHz	−14 dB @ 20 GHz	19.66	[149]
AIN	9.8	−0.68 dB @ 40 GHz	−35.8 dB @ 40 GHz	32.4	[147]
Ta_2_O_5_	32	<0.80 dB @ 30 GHz	−40 dB @ 30 GHz	19	[150]
STO	120	0.08 dB @ 10 GHz	42 dB @ 5 GHz	600	[151]

## Data Availability

Data available on request from the authors.
